# Supplementary material for: Genomic structural variation rescues a classic biological invader from a population bottleneck
Source: Sci Adv. 2026 Jul 24;12(30):eaed3656. doi: 10.1126/sciadv.aed3656 (PMC13398492; doi:10.1126/sciadv.aed3656)
Supplement: Supplementary file 1 — Figs. S1 to S5 Tables S1 to S4 Legends for data S1 to S3 References [file sciadv.aed3656_sm.pdf]

Science Advances
AAAS

Supplementary Materials for

Genomic structural variation rescues a classic biological invader from a population bottleneck

Christopher A. Osborne *et al.*

Corresponding author: Christopher A. Osborne, caosborn@buffalo.edu;
Trevor J. Krabbenhoft, tkrabben@buffalo.edu

Sci. Adv. **12**, eaed3656 (2026)
DOI: 10.1126/sciadv.aed3656

The PDF file includes:

Figs. S1 to S5
Tables S1 to S4
Legends for data S1 to S3
References

Other Supplementary Material for this manuscript includes the following:

Data S1 to S3

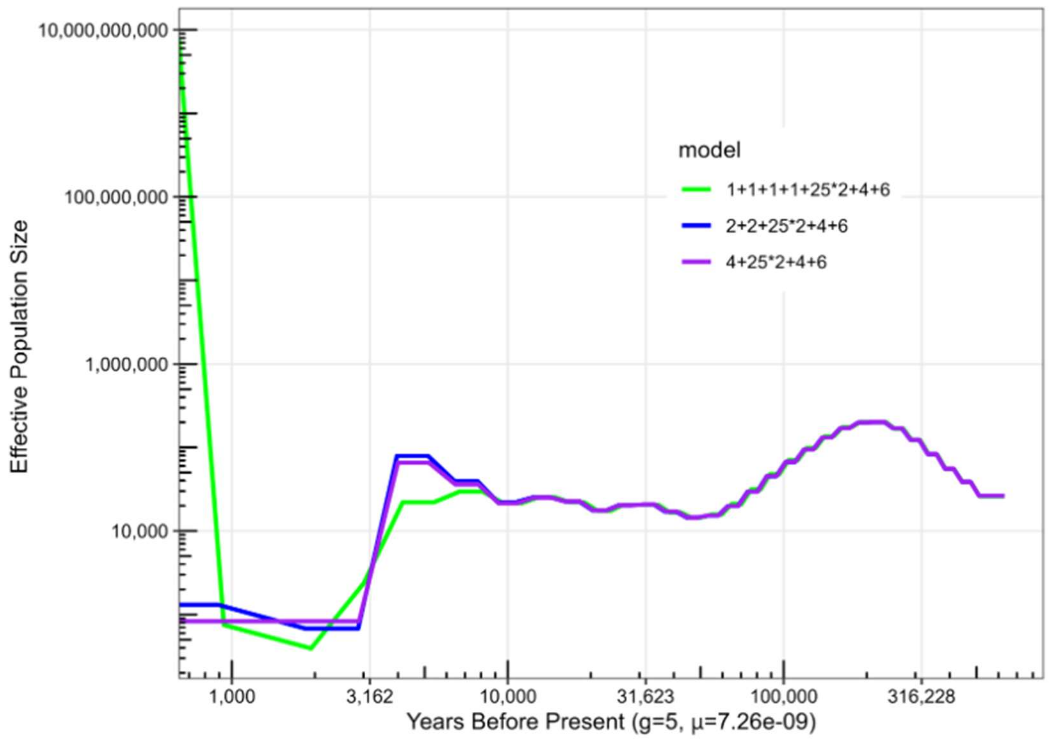

Fig. S1.

Pairwise Sequentially Markovian Coalescent (PSMC) analysis was performed to infer historical effective population size (N_e) over time. Motivated by recent findings that default PSMC time-interval parameters can generate artificial N_e peaks in the recent past (*70*), we tested three different atomic time interval (*-p*) configurations. The purple line represents the default PSMC setting (4+25*2+4+6), while the blue line represents the modified setting (2+2+25*2+4+6) recommended by the authors to resolve potential artifacts in the first time window. For this dataset, both the default and the 2+2+25*2+4+6 models are highly concordant and show no artifactual peak, indicating stable recent N_e estimates. However, highly fragmenting the most recent time intervals (1+1+1+1+25*2+4+6, green line) introduces a massive artificial spike in N_e (exceeding 10^9) at the most recent time points (<1,000 years ago), likely due to overfitting and a lack of coalescent events in such narrow time bins. All three models converge identically for historical demographic events beyond ~3,000 years ago. Axes are on a logarithmic scale, and time was scaled using a generation time (*g*) of 5 years and a mutation rate (*mu*) of 7.26e-09 per site per generation.

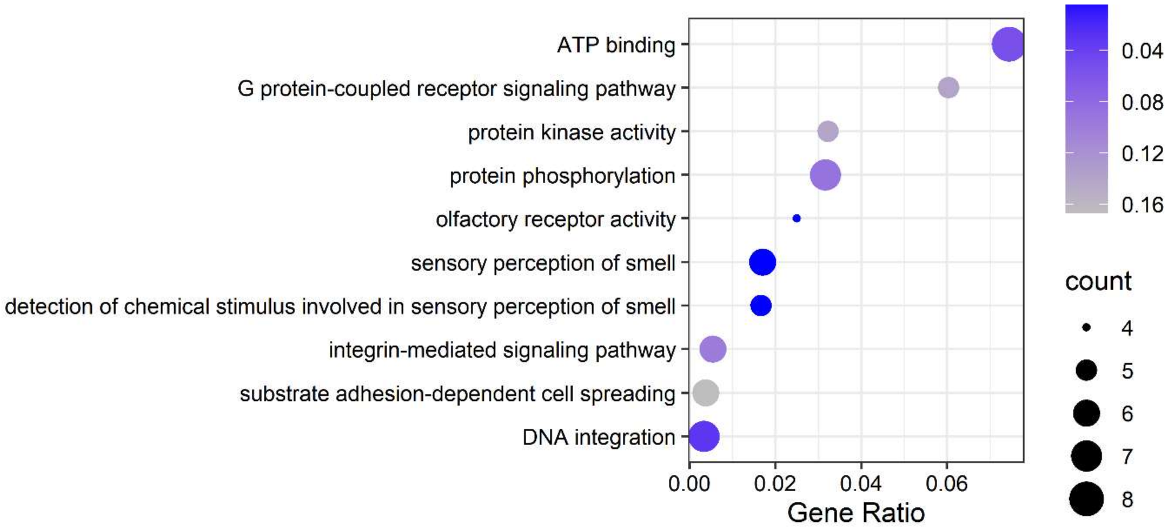

Fig. S2.

The top 10 enriched Gene Ontology terms for genes affected by genomic structural variants. Dot size corresponds to the number of SV-affected genes for each GO term.

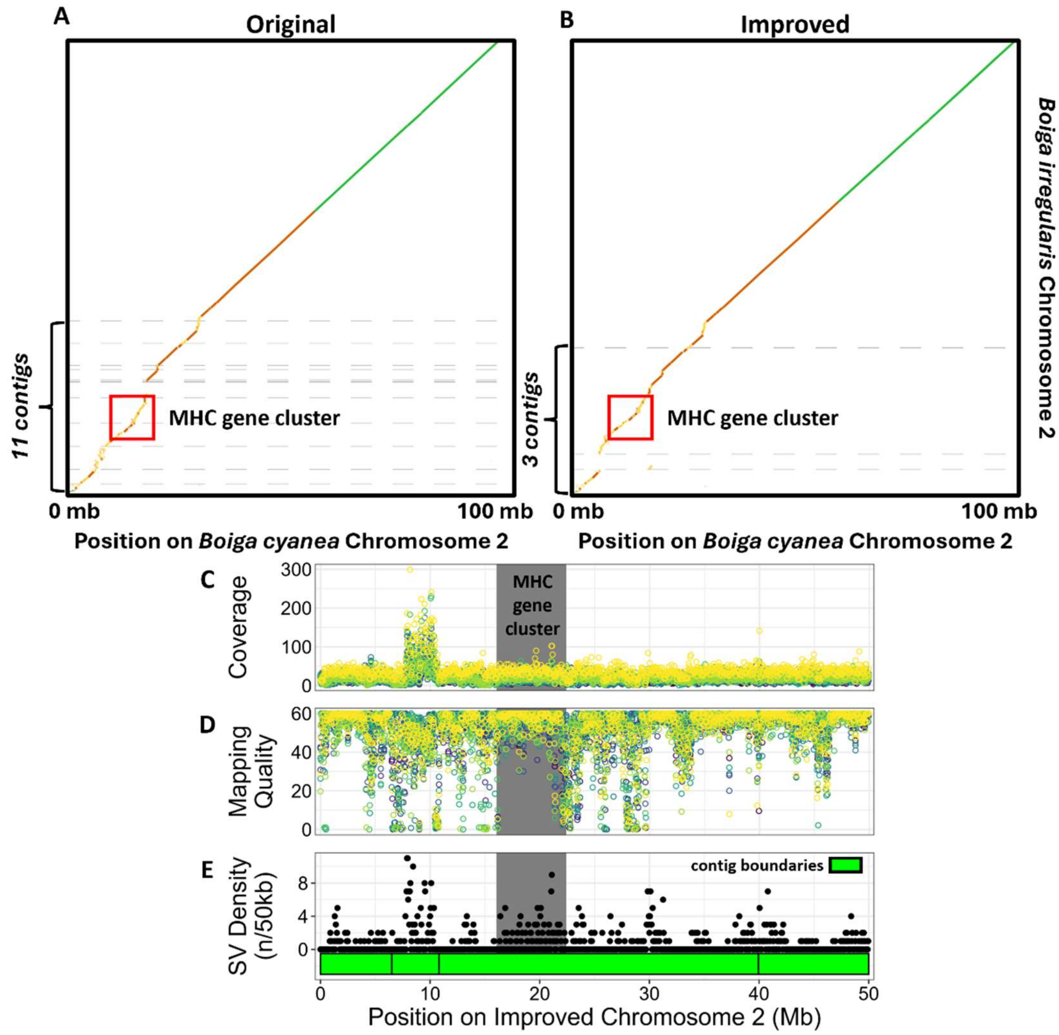

Fig. S3.

Dot plots showing synteny across the first 100 megabases (Mb) of chromosome 2 between *Boiga cyanea* and two versions of the *B. irregularis* assembly: (**A**) the original assembly scaffolded solely with Hi-C data, and (**B**) the improved assembly scaffolded with Hi-C data plus reference-guided assembly using *B. cyanea*. Line colors indicate relative sequence identity (green: >50%; orange: 25–50%; yellow: <25%). Dashed horizontal lines indicate contig breakpoints in both *B. irregularis* assemblies. (**C** to **E**) Sequence quality metrics mapped across the improved chromosome 2 assembly. (**C**) Coverage and (**D**) mapping quality scores of Oxford Nanopore Technology (ONT) long-read sequences for eight *B. irregularis* samples (differentiated by point color). Points represent averages calculated in non-overlapping 50-kilobase (kb) windows. (**E**) Density of structural variants (SVs), expressed as the number of SVs per 50-kb window. Green blocks at the bottom denote contig boundaries in the improved assembly. Across all panels, the location of a major histocompatibility complex (MHC) gene cluster is highlighted (red boxes in **A** and **B**; vertical grey shaded region in **C** through **E**).

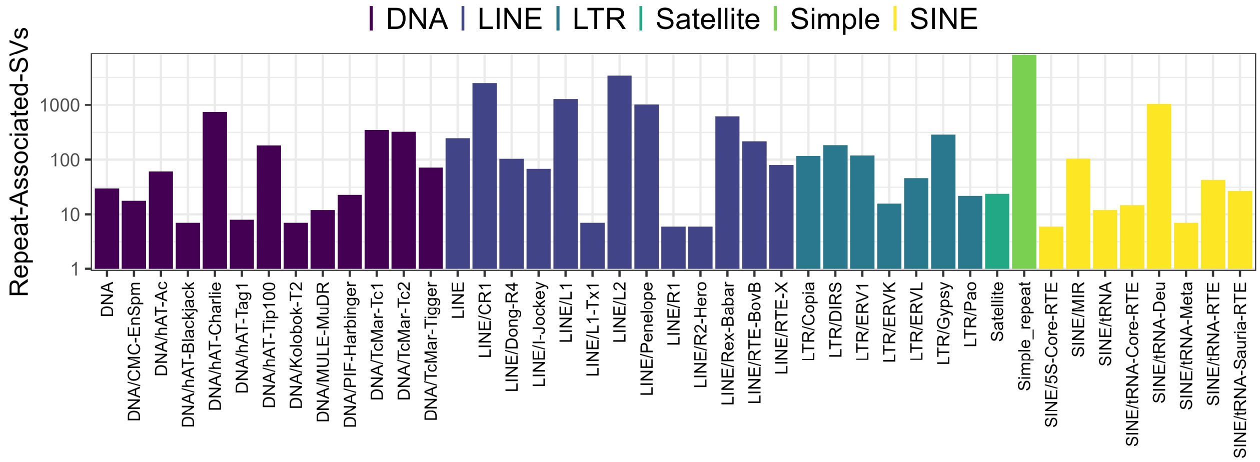

Fig. S4.

Bar chart displaying the number of repeats found to be associated with structural variants (SV). Repeats found within two or fewer SVs are displayed. Bars are colored by repeat class.

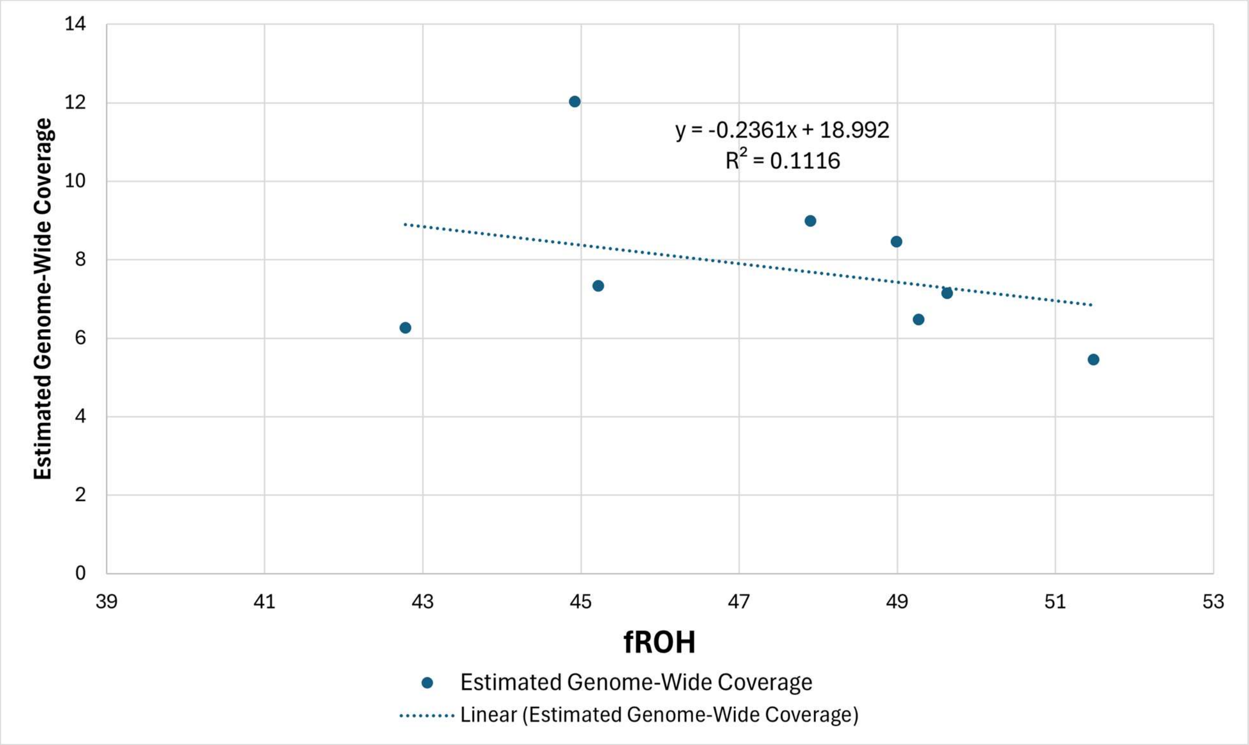

Fig. S5.

Relationship between estimated genome-wide sequencing coverage and the proportion of the genome in runs of homozygosity (fROH) in *Boiga irregularis* (brown treesnake) resequencing samples. Each point represents an individual, illustrating how sequencing depth influences the detection of homozygous regions across the genome.

Family	Scientific Name	Scaffold N50 (Mb)	Contig N50 (Mb)	Genome Accession/ Version	Citation
Typhlopidae	*Argyrophis diardii*	265.0	25.6	NA	(*87*)
Xenopeltidae	*Xenopeltis unicolor*	199.5	57.0	NA	(*87*)
Erycidae	*Eryx tataricus*	210.2	17.6	NA	(*87*)
Cylindrophiidae	*Cylindrophis ruffus*	204.3	47.3	NA	(*87*)
Xenodermatidae	*Achalinus jinggangensis*	226.4	8.3	NA	(*87*)
Colubridae	*Pantherophis guttatus*	146.9	26.7	NA	(*87*)
Pareatidae	*Pareas berdmorei*	144.0	13.5	NA	(*87*)
Lamprophiidae	*Psammodynastes pulverulentus*	217.0	32.2	NA	(*87*)
Viperidae	*Gloydius shedaoensis*	191.5	6.7	NA	(*87*)
Lamprophiidae	*Boaedon fuliginosus*	159.2	24.9	NA	(*87*)
Elapidae	*Naja naja*	224.1	0.3	GCA_009733165.1	(*161*)
Elapidae	*Bungarus multicinctus*	135.4	14.5	CNA0045869	(*162*)
Dipsadidae	*Thermophis baileyi*	139.9	4.0	GWHBJWY00000000	(*163*)
Calamariidae	*Calamaria septentrionalis*	249.1	13.5	NA	(*87*)
Colubridae	*Ahaetulla prasina*	232.0	23.9	GWHBRAF00000000	(*164*)
Colubridae	*Thamnophis elegans*	100.9	4.6	GCF_009769535.1	NA

Table S1.

The 16 chromosome-level snake genomes used for synteny-based sex chromosome identification and as evidence for genome annotations in this study.

Sample	Number of ROH	Average ROH Length	Cumulative ROH Length	fROH	Length Of Largest ROH
**F1**	337	2,650,445.10	893,200,000	63.1	143,200,000
**F2**	1067	595,520.44	635,420,311	44.9	7,665,858
**F3**	1229	551,276.13	677,518,360	47.9	11,427,867
**F4**	1141	530,399.91	605,186,296	42.8	12,882,412
**M1**	1247	584,049.59	728,309,839	51.5	11,050,096
**M2**	1111	631,906.50	702,048,122	49.6	12,269,865
**M3**	1090	586,857.67	639,674,855	45.2	7,803,483
**M4**	1207	574,126.37	692,970,523	49.0	9,514,386
**M5**	1276	546,276.17	697,048,388	49.3	10,584,963

Table S2.

Table showing ROH characteristics for nine *Boiga irregularis* samples used in this study.

Sample	Read Count	Number of Bases (Gb)	Coverage	Read N50	Mean RL	Coverage of UL reads (>50 Kb)	Mean Q-score
**F1**	4,401,467	74.498	42.60	26,959	16,925	2.4	20.9
**F2**	971,571	19.854	12.03	30,183	20,435	1.3	23.3
**F3**	806,141	10.339	6.26	33,972	12,826	1.4	22.7
**F4**	1,460,735	14.844	8.99	25,664	10,162	1.5	23.3
**M1**	500,190	8.999	5.45	41,251	17,991	1.9	23.4
**M2**	749,290	11.802	7.15	36,738	15,751	2.1	23.4
**M3**	764,892	12.096	7.33	28,082	15,814	0.7	23.2
**M4**	753,081	13.971	8.46	37,011	18,551	2.0	23.3
**M5**	638,968	10.694	6.48	34,292	16,737	1.4	23.4

Table S3.

Table showing quality characteristics of Oxford Nanopore Technologies long-read sequencing data for the nine *B. irregularis* samples used in this study.

Sample ID	Location	Latitude	Longitude	Collection Year	Use
M1	Guam	13.574866	144.932499	2025	Resequencing
M2	Guam	13.574866	144.932499	2025	Resequencing
M3	Guam	13.574866	144.932499	2025	Resequencing
M4	Guam	13.574866	144.932499	2025	Resequencing
M5	Guam	13.574866	144.932499	2025	Resequencing
F1	Cocos Island	13.239978	144.654028	2021	Genome Assembly
F2	Guam	13.574866	144.932499	2025	Resequencing
F3	Guam	13.574866	144.932499	2025	Resequencing
F4	Guam	13.574866	144.932499	2025	Resequencing

Table S4.

Table showing the location and collection date for the nine *Boiga irregularis* samples used in this study.

Data S1. (separate file)

Gene ontology enrichment table for genes found within runs of homozygosity in three or fewer individuals (ROH deserts) and found within runs of homozygosity seven or more individuals (ROH islands).

Data S2. (separate file)

Data on the genomic location and functional annotation results of genes from the Major Histocompatibility Complex (MHC) locus in *Boiga irregularis*. Additional tabs include a list and the locations of MHC genes found to be affected by SVs.

Data S3. (separate file)

Data on the genomic location and functional annotation results of type-II vomeronasal receptors (V2R) genes in *Boiga irregularis*. Additional tabs include a list and the locations of V2R genes found to be affected by SVs and those contained within ROH Deserts.

REFERENCES

1. M. Lynch, J. Conery, R. Burger, Mutation accumulation and the extinction of small populations. *Am. Nat.* **146**, 489–518 (1995).

2. R. Lande, S. Shannon, The role of genetic variation in adaptation and population persistence in a changing environment. *Evolution* **50**, 434–437 (1996).

3. G. Booy, R. J. J. Hendriks, M. J. M. Smulders, J. M. Van Groenendael, B. Vosman, Genetic diversity and the survival of populations. *Plant Biol.* **2**, 379–395 (2000).

4. E. Mayr, *Animal Species and Evolution* (Harvard Univ. Press, 1963).

5. R. C. Lacy, Importance of genetic variation to the viability of mammalian populations. *J. Mammal.* **78**, 320–335 (1997).

6. D. H. Reed, R. Frankham, Correlation between fitness and genetic diversity. *Conserv. Biol.* **17**, 230–237 (2003).

7. R. Lande, Genetics and demography in biological conservation. *Science* **241**, 1455–1460 (1988).

8. K. A. Crandall, O. R. P. Bininda-Emonds, G. M. Mace, R. K. Wayne, Considering evolutionary processes in conservation biology. *Trends Ecol. Evol.* **15**, 290–295 (2000).

9. R. Frankham, Genetics and extinction. *Biol. Conserv.* **126**, 131–140 (2005).

10. M. H. Kohn, W. J. Murphy, E. A. Ostrander, R. K. Wayne, Genomics and conservation genetics. *Trends Ecol. Evol.* **21**, 629–637 (2006).

11. F. W. Allendorf, P. A. Hohenlohe, G. Luikart, Genomics and the future of conservation genetics. *Nat. Rev. Genet.* **11**, 697–709 (2010).

12. J. A. DeWoody, A. M. Harder, S. Mathur, J. R. Willoughby, The long-standing significance of genetic diversity in conservation. *Mol. Ecol.* **30**, 4147–4154 (2021).

13. M. Kardos, E. E. Armstrong, S. W. Fitzpatrick, S. Hauser, P. W. Hedrick, J. M. Miller, D. A. Tallmon, W. C. Funk, The crucial role of genome-wide genetic variation in conservation. *Proc. Natl. Acad. Sci. U.S.A.* **118**, e2104642118 (2021).

14. J. R. Chapman, S. Nakagawa, D. W. Coltman, J. Slate, B. C. Sheldon, A quantitative review of heterozygosity–fitness correlations in animal populations. *Mol. Ecol.* **18**, 2746–2765 (2009).

15. J. L. A. Wood, M. C. Yates, D. J. Fraser, Are heritability and selection related to population size in nature? Meta-analysis and conservation implications. *Evol. Appl.* **9**, 640–657 (2016).

16. J. C. Teixeira, C. D. Huber, The inflated significance of neutral genetic diversity in conservation genetics. *Proc. Natl. Acad. Sci. U.S.A.* **118**, e2015096118 (2021).

17. N. Dussex, H. E. Morales, C. Grossen, L. Dalén, C. Van Oosterhout, Purging and accumulation of genetic load in conservation. *Trends Ecol. Evol.* **38**, 961–969 (2023).

18. D. H. Reed, Albatrosses, eagles and newts, Oh My!: Exceptions to the prevailing paradigm concerning genetic diversity and population viability? *Anim. Conserv.* **13**, 448–457 (2010).

19. J. A. Robinson, D. Ortega-Del Vecchyo, Z. Fan, B. Y. Kim, B. M. vonHoldt, C. D. Marsden, K. E. Lohmueller, R. K. Wayne, Genomic Flatlining in the Endangered Island Fox. *Curr. Biol.* **26**, 1183–1189 (2016).

20. M. V. Westbury, S. Hartmann, A. Barlow, I. Wiesel, V. Leo, R. Welch, D. M. Parker, F. Sicks, A. Ludwig, L. Dalén, M. Hofreiter, Extended and continuous decline in effective population size results in low genomic diversity in the world's rarest hyena species, the brown hyena. *Mol. Biol. Evol.* **35**, 1225–1237 (2018).

21. M. C. Yates, E. Bowles, D. J. Fraser, Small population size and low genomic diversity have no effect on fitness in experimental translocations of a wild fish. *Proc. R. Soc. B* **286**, 20191989 (2019).

22. P. A. Morin, F. I. Archer, C. D. Avila, J. R. Balacco, Y. V. Bukhman, W. Chow, O. Fedrigo, G. Formenti, J. A. Fronczek, A. Fungtammasan, F. M. D. Gulland, B. Haase, M. Peter Heide-

Jorgensen, M. L. Houck, K. Howe, A. C. Misuraca, J. Mountcastle, W. Musser, S. Paez, S. Pelan, A. Phillippy, A. Rhie, J. Robinson, L. Rojas-Bracho, T. K. Rowles, O. A. Ryder, C. R. Smith, S. Stevenson, B. L. Taylor, J. Teilmann, J. Torrance, R. S. Wells, A. J. Westgate, E. D. Jarvis, Reference genome and demographic history of the most endangered marine mammal, the vaquita. *Mol. Ecol. Resour.* **21**, 1008–1020 (2021).

23. D. Kleinman-Ruiz, M. Lucena-Perez, B. Villanueva, J. Fernández, A. P. Saveljev, M. Ratkiewicz, K. Schmidt, N. Galtier, A. García-Dorado, J. A. Godoy, Purging of deleterious burden in the endangered Iberian lynx. *Proc. Natl. Acad. Sci. U.S.A.* **119**, e2110614119 (2022).

24. H. Ellegren, G. Hartman, M. Johansson, L. Andersson, Major histocompatibility complex monomorphism and low levels of DNA fingerprinting variability in a reintroduced and rapidly expanding population of beavers. *Proc. Natl. Acad. Sci. U.S.A.* **90**, 8150–8153 (1993).

25. B. Epstein, M. Jones, R. Hamede, S. Hendricks, H. McCallum, E. P. Murchison, B. Schönfeld, C. Wiench, P. Hohenlohe, A. Storfer, Rapid evolutionary response to a transmissible cancer in Tasmanian devils. *Nat. Commun.* **7**, 12684 (2016).

26. J. R. Willoughby, A. M. Harder, J. A. Tennessen, K. T. Scribner, M. R. Christie, Rapid genetic adaptation to a novel environment despite a genome-wide reduction in genetic diversity. *Mol. Ecol.* **27**, 4041–4051 (2018).

27. G. G. Auteri, L. L. Knowles, Decimated little brown bats show potential for adaptive change. *Sci. Rep.* **10**, 3023 (2020).

28. H.-B. Jeon, M. Yates, B. K. Gallagher, D. J. Fraser, Life's a ditch: Demographic history and environmental factors shape fine-scale local adaptation within small populations of brook trout. *Can. J. Fish. Aquat. Sci.* **82**, 1 (2025).

29. A. D. Yoder, J. W. Poelstra, G. P. Tiley, R. C. Williams, Neutral theory is the foundation of conservation genetics. *Mol. Biol. Evol.* **35**, 1322–1326 (2018).

30. J. Olano-Marin, J. C. Mueller, B. Kempenaers, Heterozygosity and survival in blue tits (*Cyanistes caeruleus*): Contrasting effects of presumably functional and neutral loci. *Mol. Ecol.* **20**, 4028–4041 (2011).

31. R. Holderegger, U. Kamm, F. Gugerli, Adaptive vs. neutral genetic diversity: Implications for landscape genetics. *Landsc. Ecol.* **21**, 797–807 (2006).

32. S. Mathur, A. J. Mason, G. S. Bradburd, H. L. Gibbs, Functional genomic diversity is correlated with neutral genomic diversity in populations of an endangered rattlesnake. *Proc. Natl. Acad. Sci. U.S.A.* **120**, e2303043120 (2023).

33. D. N. Anstett, J. Anstett, S. N. Sheth, D. R. Moxley, M. Jahani, K. Huang, M. Todesco, R. Jordan, J. M. Lazaro-Guevara, L. H. Rieseberg, A. L. Angert, Evolutionary rescue during extreme drought. bioRxiv 619808 [Preprint] (2024).

34. P. Nosil, D. J. Funk, D. Ortiz-Barrientos, Divergent selection and heterogeneous genomic divergence. *Mol. Ecol.* **18**, 375–402 (2009).

35. D. J. Prince, S. M. O'Rourke, T. Q. Thompson, O. A. Ali, H. S. Lyman, I. K. Saglam, T. J. Hotaling, A. P. Spidle, M. R. Miller, The evolutionary basis of premature migration in Pacific salmon highlights the utility of genomics for informing conservation. *Sci. Adv.* **3**, e1603198 (2017).

36. K. Huang, L. H. Rieseberg, Frequency, origins, and evolutionary role of chromosomal inversions in plants. *Front. Plant Sci.* **11**, 296 (2020).

37. K. Stenløkk, M. Saitou, L. Rud-Johansen, T. Nome, M. Moser, M. Árnyasi, M. Kent, N. J. Barson, S. Lien, The emergence of supergenes from inversions in Atlantic salmon. *Philos. Trans. R Soc Lond. B Biol. Sci.* **377**, 20210195 (2022).

38. M. Jamsandekar, M. S. Ferreira, M. E. Pettersson, E. D. Farrell, B. W. Davis, L. Andersson, The origin and maintenance of supergenes contributing to ecological adaptation in Atlantic herring. *Nat. Commun.* **15**, 9136 (2024).

39. O. S. Harringmeyer, H. E. Hoekstra, Massive inversion polymorphisms shape the genomic landscape of deer mice. *Nat. Ecol Evol.* **6**, 1965–1979 (2022).

40. C. Mérot, R. A. Oomen, A. Tigano, M. Wellenreuther, A roadmap for understanding the evolutionary significance of structural genomic variation. *Trends Ecol. Evol.* **35**, 561–572 (2020).

41. D. Beyter, H. Ingimundardottir, A. Oddsson, H. P. Eggertsson, E. Bjornsson, H. Jonsson, B. A. Atlason, S. Kristmundsdottir, S. Mehringer, M. T. Hardarson, S. A. Gudjonsson, D. N. Magnusdottir, A. Jonasdottir, A. Jonasdottir, R. P. Kristjansson, S. T. Sverrisson, G. Holley, G. Palsson, O. A. Stefansson, G. Eyjolfsson, I. Olafsson, O. Sigurdardottir, B. Torfason, G. Masson, A. Helgason, U. Thorsteinsdottir, H. Holm, D. F. Gudbjartsson, P. Sulem, O. T. Magnusson, B. V. Halldorsson, K. Stefansson, Long-read sequencing of 3,622 Icelanders provides insight into the role of structural variants in human diseases and other traits. *Nat. Genet.* **53**, 779–786 (2021).

42. W. De Coster, M. H. Weissensteiner, F. J. Sedlazeck, Towards population-scale long-read sequencing. *Nat. Rev. Genet.* **22**, 572–587 (2021).

43. E. J. Hollox, L. W. Zuccherato, S. Tucci, Genome structural variation in human evolution. *Trends Genet.* **38**, 45–58 (2022).

44. A. W. Khan, V. Garg, M. Roorkiwal, A. A. Golicz, D. Edwards, R. K. Varshney, Super-pangenome by integrating the wild side of a species for accelerated crop improvement. *Trends Plant Sci.* **25**, 148–158 (2020).

45. Y. Zhou, Z. Zhang, Z. Bao, H. Li, Y. Lyu, Y. Zan, Y. Wu, L. Cheng, Y. Fang, K. Wu, J. Zhang, H. Lyu, T. Lin, Q. Gao, S. Saha, L. Mueller, Z. Fei, T. Städler, S. Xu, Z. Zhang, D. Speed, S. Huang, Graph pangenome captures missing heritability and empowers tomato breeding. *Nature* **606**, 527–534 (2022).

46. J. Wold, S. Galla, D. Eccles, C. J. Hogg, K.-P. Koepfli, M. L. Lec, J. Guhlin, K. Price, J. Roberts, T. Steeves, Expanding the conservation genomics toolbox: Incorporating structural

variants to enhance genomic studies for species of conservation concern. *Mol. Ecol.* **30**, 5949–5965 (2021).

47. J. Stapley, A. W. Santure, S. R. Dennis, Transposable elements as agents of rapid adaptation may explain the genetic paradox of invasive species. *Mol. Ecol.* **24**, 2241–2252 (2015).

48. H. De Kort, S. Legrand, O. Honnay, J. Buckley, Transposable elements maintain genome-wide heterozygosity in inbred populations. *Nat. Commun.* **13**, 7022 (2022).

49. C. J. VanGessel, T. J. Felderhoff, D. M. Prigozhin, M. Cui, G. Pressoir, A. L. Healey, J. T. Lovell, V. J. Nalam, M. T. Nishimura, G. P. Morris, Ancient pangenomic origins of noncanonical NLR genes underlying the recent evolutionary rescue of a staple crop. bioRxiv 648396 [Preprint] (2025).

50. J. Wilson, V. C. Bieker, L. V. Boheemen, T. Connallon, M. D. Martin, P. Battlay, K. A. Hodgins, Copy number variation contributes to parallel local adaptation in an invasive plant. *Proc. Natl. Acad. Sci. U.S.A.* **122**, e2413587122 (2025).

51. S. Secomandi, G. R. Gallo, R. Rossi, C. Rodríguez Fernandes, E. D. Jarvis, A. Bonisoli-Alquati, L. Gianfranceschi, G. Formenti, Pangenome graphs and their applications in biodiversity genomics. *Nat. Genet.* **57**, 13–26 (2025).

52. T. M. Pegan, A. H. Sivakumar, C. A. Burns, K. A. Lopez, B. Fang, N. M. Melendez, L. Bartel, S. V. Edwards, The value of structural variants to conservation genomics in the pangenome era. *J. Hered.* **esaf098**, (2025).

53. P. J. Prentis, J. R. U. Wilson, E. E. Dormontt, D. M. Richardson, A. J. Lowe, Adaptive evolution in invasive species. *Trends Plant Sci.* **13**, 288–294 (2008).

54. D. G. Bock, C. Caseys, R. D. Cousens, M. A. Hahn, S. M. Heredia, S. Hübner, K. G. Turner, K. D. Whitney, L. H. Rieseberg, "What we still don't know about invasion genetics" in *Invasion Genetics*, S. C. H. Barrett, R. I. Colautti, K. M. Dlugosch, L. H. Rieseberg, Eds. (Wiley, ed. 1, 2016), pp. 346–370; https://onlinelibrary.wiley.com/doi/10.1002/9781119072799.ch20.

55. S. Sherpa, L. Després, The evolutionary dynamics of biological invasions: A multi-approach perspective. *Evol. Appl.* **14**, 1463–1484 (2021).

56. M. M. Sparks, C. E. Schraidt, X. Yin, L. W. Seeb, M. R. Christie, Rapid genetic adaptation to a novel ecosystem despite a large founder event. *Mol. Ecol.* **33**, e17121 (2024).

57. S. R. Keller, D. R. Taylor, History, chance and adaptation during biological invasion: Separating stochastic phenotypic evolution from response to selection. *Ecol. Lett.* **11**, 852–866 (2008).

58. D. G. Chapple, S. M. Simmonds, B. B. M. Wong, Can behavioral and personality traits influence the success of unintentional species introductions? *Trends Ecol. Evol.* **27**, 57–64 (2012).

59. S. J. Novak, The role of evolution in the invasion process. *Proc. Natl. Acad. Sci. U.S.A.* **104**, 3671–3672 (2007).

60. T. H. Fritts, G. H. Rodda, The role of introduced species in the degradation of island ecosystems: A case history of Guam. *Annu. Rev. Ecol. Syst.* **29**, 113–140 (1998).

61. G. H. Rodda, T. H. Fritts, P. J. Conry, Origin and population growth of the brown tree snake, *Boiga irregularis*, on Guam! *Pac. Sci.* **13**, 47–56 (1992).

62. G. J. Wiles, J. Bart, R. E. Beck, C. F. Aguon, Impacts of the brown tree snake: Patterns of decline and species persistence in Guam's avifauna. *Conserv. Biol.* **17**, 1350–1360 (2003).

63. J. Q. Richmond, D. A. Wood, J. W. Stanford, R. N. Fisher, Testing for multiple invasion routes and source populations for the invasive brown treesnake (Boiga irregularis) on Guam: Implications for pest management. *Biol. Invasions* **17**, 337–349 (2015).

64. B. A. Levine, M. R. Douglas, A. A. Yackel Adams, B. Lardner, R. N. Reed, J. A. Savidge, M. E. Douglas, Genomic pedigree reconstruction identifies predictors of mating and reproductive success in an invasive vertebrate. *Ecol. Evol.* **9**, 11863–11877 (2019).

65. Z. Wang, W. Wu, F. Shen, J.-L. Ren, C. Yan, C.-Y. Tang, X. Zhang, J.-T. Li, Genomic adaptations for tail-length evolution in arboreal snakes. *Mol. Biol. Evol.* **43**, msag029 (2026).

66. L. Singh, Evolution of karyotypes in snakes. *Chromosoma* **38**, 185–236 (1972).

67. H. Cheng, E. D. Jarvis, O. Fedrigo, K.-P. Koepfli, L. Urban, N. J. Gemmell, H. Li, Haplotype-resolved assembly of diploid genomes without parental data. *Nat. Biotechnol.* **40**, 1332–1335 (2022).

68. E. de Jong, L. Parata, P. E. Bayer, S. Corrigan, R. J. Edwards, Toward genome assemblies for all marine vertebrates: Current landscape and challenges. *GigaScience* **13**, giad119 (2024).

69. H. Li, R. Durbin, Inference of human population history from individual whole-genome sequences. *Nature* **475**, 493–496 (2011).

70. L. Hilgers, S. Liu, A. Jensen, T. Brown, T. Cousins, R. Schweiger, K. Guschanski, M. Hiller, Avoidable false PSMC population size peaks occur across numerous studies. *Curr. Biol.* **35**, 927–930.e3 (2025).

71. N. Mather, S. M. Traves, S. Y. W. Ho, A practical introduction to sequentially Markovian coalescent methods for estimating demographic history from genomic data. *Ecol. Evol.* **10**, 579–589 (2020).

72. E. Santiago, C. Köpke, A. Caballero, Accounting for population structure and data quality in demographic inference with linkage disequilibrium methods. *Nat. Commun.* **16**, 6054 (2025).

73. C. Simkanin, J. T. Carlton, B. Steves, P. Fofonoff, J. C. Nelson, C. Clarke Murray, G. M. Ruiz, Exploring potential establishment of marine rafting species after transoceanic long-distance dispersal. *Glob. Ecol. Biogeogr.* **28**, 588–600 (2019).

74. F. C. Ceballos, P. K. Joshi, D. W. Clark, M. Ramsay, J. F. Wilson, Runs of homozygosity: Windows into population history and trait architecture. *Nat. Rev. Genet.* **19**, 220–234 (2018).

75. M. Kirin, R. McQuillan, C. S. Franklin, H. Campbell, P. M. McKeigue, J. F. Wilson, Genomic runs of homozygosity record population history and consanguinity. *PLOS ONE* **5**, e13996 (2010).

76. G. Renaud, K. Hanghøj, T. S. Korneliussen, E. Willerslev, L. Orlando, Joint estimates of heterozygosity and runs of homozygosity for modern and ancient samples. *Genetics* **212**, 587–614 (2019).

77. M. A. Stoffel, S. E. Johnston, J. G. Pilkington, J. M. Pemberton, Genetic architecture and lifetime dynamics of inbreeding depression in a wild mammal. *Nat. Commun.* **12**, 2972 (2021).

78. M. Kardos, G. Luikart, F. W. Allendorf, Measuring individual inbreeding in the age of genomics: Marker-based measures are better than pedigrees. *Heredity* **115**, 63–72 (2015).

79. S. Romain, C. Lemaitre, SVJedi-graph: Improving the genotyping of close and overlapping structural variants with long reads using a variation graph. *Bioinformatics* **39**, i270–i278 (2023).

80. E. A. O'Connor, H. Westerdahl, R. Burri, S. V. Edwards, Avian MHC evolution in the era of genomics: Phase 1.0. *Cells* **8**, 1152 (2019).

81. K. He, P. Minias, P. O. Dunn, Long-read genome assemblies reveal extraordinary variation in the number and structure of MHC loci in birds. *Genome Biol. Evol.* **13**, evaa270 (2021).

82. A. T. Dilthey, State-of-the-art genome inference in the human MHC. *Int. J. Biochem. Cell Biol.* **131**, 105882 (2021).

83. T. Pšenička, B. Augstenová, D. Frynta, P. Kornilios, L. Kratochvíl, M. Rovatsos, Sex chromosome turnovers and stability in snakes. *Mol. Biol. Evol.* **42**, msae255 (2025).

84. A. Ochoa, H. L. Gibbs, Genomic signatures of inbreeding and mutation load in a threatened rattlesnake. *Mol. Ecol.* **30**, 5454–5496 (2021).

85. J. A. Savidge, F. J. Qualls, G. H. Rodda, Reproductive biology of the brown tree snake, *Boiga irregularis* (Reptilia: Colubridae), during colonization of Guam and comparison with that in their native range[1]. *Pac. Sci.* **61**, 191–199 (2007).

86. T. Mathies, J. A. Cruz, V. A. Lance, J. A. Savidge, Reproductive biology of male brown treesnakes (*Boiga irregularis*) on Guam. *J. Herpetol.* **44**, 209–221 (2010).

87. S. A. Martin, G. J. Lipps Jr., H. L. Gibbs, Pedigree-based assessment of recent population connectivity in a threatened rattlesnake. *Mol. Ecol. Resour.* **21**, 1820–1832 (2021).

88. A. Castro-Prieto, B. Wachter, S. Sommer, Cheetah paradigm revisited: MHC diversity in the world's largest free-ranging population. *Mol. Biol. Evol.* **28**, 1455–1468 (2011).

89. A. Ellison, J. Allainguillaume, S. Girdwood, J. Pachebat, K. M. Peat, P. Wright, S. Consuegra, Maintaining functional major histocompatibility complex diversity under inbreeding: The case of a selfing vertebrate. *Proc. R. Soc. B* **279**, 5004–5013 (2012).

90. D. J. Penn, K. Damjanovich, W. K. Potts, MHC heterozygosity confers a selective advantage against multiple-strain infections. *Proc. Natl. Acad. Sci. U.S.A.* **99**, 11260–11264 (2002).

91. S. Prost, A. P. Machado, J. Zumbroich, L. Preier, S. Mahtani-Williams, R. Meissner, K. Guschanski, J. C. Brealey, C. R. Fernandes, P. Vercammen, L. T. B. Hunter, A. V. Abramov, M. Plasil, P. Horin, L. Godsall-Bottriell, P. Bottriell, D. L. Dalton, A. Kotze, P. A. Burger, Genomic analyses show extremely perilous conservation status of African and Asiatic cheetahs (*Acinonyx jubatus*). *Mol. Ecol.* **31**, 4208–4223 (2022).

92. Y. Tang, G. Liu, S. Zhao, K. Li, D. Zhang, S. Liu, D. Hu, Major histocompatibility complex (MHC) diversity of the reintroduction populations of endangered Przewalski's horse. *Genes* **13**, 928 (2022).

93. C. P. Jaeger, M. R. Duvall, B. J. Swanson, C. A. Phillips, M. J. Dreslik, S. J. Baker, R. B. King, Microsatellite and major histocompatibility complex variation in an endangered rattlesnake, the Eastern Massasauga (*Sistrurus catenatus*). *Ecol. Evol.* **6**, 3991–4003 (2016).

94. T. Kamiya, K. O'Dwyer, H. Westerdahl, A. Senior, S. Nakagawa, A quantitative review of MHC-based mating preference: The role of diversity and dissimilarity. *Mol. Ecol.* **23**, 5151–5163 (2014).

95. J. C. Winternitz, S. G. Minchey, L. Z. Garamszegi, S. Huang, P. R. Stephens, S. Altizer, Sexual selection explains more functional variation in the mammalian major histocompatibility complex than parasitism. *Proc. Biol. Sci.* **280**, 20131605 (2013).

96. N. Schwensow, M. Eberle, S. Sommer, Compatibility counts: MHC-associated mate choice in a wild promiscuous primate. *Proc. Biol. Sci.* **275**, 555–564 (2008).

97. D. J. Penn, W. K. Potts, The evolution of mating preferences and major histocompatibility complex genes. *Am. Nat.* **153**, 145–164 (1999).

98. S. Leclaire, M. Strandh, J. Mardon, H. Westerdahl, F. Bonadonna, Odour-based discrimination of similarity at the major histocompatibility complex in birds. *Proc. R. Soc. B* **284**, 20162466 (2017).

99. T. Leinders-Zufall, P. Brennan, P. Widmayer, S. Prashanth Chandramani, A. Maul-Pavicic, M. Jäger, X.-H. Li, H. Breer, F. Zufall, T. Boehm, MHC class I peptides as chemosensory signals in the vomeronasal organ. *Science* **306**, 1033–1037 (2004).

100. T. Leinders-Zufall, T. Ishii, P. Mombaerts, F. Zufall, T. Boehm, Structural requirements for the activation of vomeronasal sensory neurons by MHC peptides. *Nat. Neurosci.* **12**, 1551–1558 (2009).

101. U. Brykczynska, A. C. Tzika, I. Rodriguez, M. C. Milinkovitch, Contrasted evolution of the vomeronasal receptor repertoires in mammals and squamate reptiles. *Genome Biol. Evol.* **5**, 389–401 (2013).

102. J. P. Elbers, S. S. Taylor, Major histocompatibility complex polymorphism in reptile conservation. *Herpetol. Conserv. Biol.* **1**, 1–12 (2016).

103. Q.-H. Han, R.-N. Sun, H.-Q. Yang, Z.-W. Wang, Q.-H. Wan, S.-G. Fang, MHC class I diversity predicts non-random mating in Chinese alligators (Alligator sinensis). *Heredity* **122**, 809–818 (2019).

104. H. C. Miller, J. A. Moore, N. J. Nelson, C. H. Daugherty, Influence of major histocompatibility complex genotype on mating success in a free-ranging reptile population. *Proc. Biol. Sci.* **276**, 1695–1704 (2009).

105. M. Olsson, T. Madsen, J. Nordby, E. Wapstra, B. Ujvari, H. Wittsell, Major histocompatibility complex and mate choice in sand lizards. *Proc. Biol. Sci.* **270**, S254–S256 (2003).

106. M. A. Brockhurst, E. Harrison, J. P. J. Hall, T. Richards, A. McNally, C. MacLean, The ecology and evolution of pangenomes. *Curr. Biol.* **29**, R1094–R1103 (2019).

107. V. Buffalo, Quantifying the relationship between genetic diversity and population size suggests natural selection cannot explain Lewontin's Paradox. *eLife* **10**, e67509 (2021).

108. B. Fang, S. V. Edwards, Pangenomes: New tools for ecological and evolutionary genomics. *Trends Ecol. Evol.* **41**, 230–244 (2026).

109. M. D. Roberts, E. B. Josephs, *k*-mer-based diversity scales with population size proxies more than nucleotide diversity in a meta-analysis of 98 plant species. *Evol. Lett.* **9**, 434–445 (2025).

110. R. C. Lewontin, *The Gentic Basis of Evolutionary Change* (Columbia Univ. Press, 1974).

111. C. Hoge, M. De Manuel, M. Mahgoub, N. Okami, Z. Fuller, S. Banerjee, Z. Baker, M. McNulty, P. Andolfatto, T. S. Macfarlan, M. Schumer, A. C. Tzika, M. Przeworski, Patterns of recombination in snakes reveal a tug-of-war between PRDM9 and promoter-like features. *Science* **383**, eadj7026 (2024).

112. M. Dubin, J. Fuchs, R. Gräf, I. Schubert, W. Nellen, Dynamics of a novel centromeric histone variant CenH3 reveals the evolutionary ancestral timing of centromere biogenesis. *Nucleic Acids Res.* **38**, 7526–7537 (2010).

113. E. P. Westeen, M. Escalona, E. Beraut, M. P. A. Marimuthu, O. Nguyen, R. N. Fisher, E. Toffelmier, H. B. Shaffer, I. J. Wang, A reference genome assembly for the continentally distributed ring-necked snake*, Diadophis punctatus*. *J. Hered.* **114**, 690–697 (2023).

114. D. A. Wood, J. Q. Richmond, M. Escalona, M. P. A. Marimuthu, O. Nguyen, S. Sacco, E. Beraut, M. Westphal, R. N. Fisher, A. G. Vandergast, E. Toffelmier, I. J. Wang, H. B. Shaffer, Reference genome of the California glossy snake, *Arizona elegans occidentalis* : A declining California Species of Special Concern. *J. Hered.* **113**, 632–640 (2022).

115. S. Ohno, *Sex Chromosomes and Sex-Linked Genes* (Monographs on Endocrinology Series, Springer Berlin/Heidelberg, 1967).

116. K. W. Jones, L. Singh, Snakes and the evolution of sex chromosomes. *Trends Genet.* **1**, 55–61 (1985).

117. D. Bachtrog, The temporal dynamics of processes underlying Y chromosome degeneration. *Genetics* **179**, 1513–1525 (2008).

118. B. Charlesworth, D. Charlesworth, Rapid fixation of deleterious alleles can be caused by Muller's ratchet. *Genet. Res.* **70**, 63–73 (1997).

119. Y. H. Liu, C. Luo, S. G. Golding, J. B. Ioffe, X. M. Zhou, Tradeoffs in alignment and assembly-based methods for structural variant detection with long-read sequencing data. *Nat. Commun.* **15**, 2447 (2024).

120. H. M. Schilbert, A. Rempel, B. Pucker, Comparison of read mapping and variant calling tools for the analysis of plant NGS data. *Plants* **9**, 439 (2020).

121. A. M. Wenger, P. Peluso, W. J. Rowell, P.-C. Chang, R. J. Hall, G. T. Concepcion, J. Ebler, A. Fungtammasan, A. Kolesnikov, N. D. Olson, A. Töpfer, M. Alonge, M. Mahmoud, Y. Qian, C.-S. Chin, A. M. Phillippy, M. C. Schatz, G. Myers, M. A. DePristo, J. Ruan, T. Marschall, F. J. Sedlazeck, J. M. Zook, H. Li, S. Koren, A. Carroll, D. R. Rank, M. W. Hunkapiller, Accurate circular consensus long-read sequencing improves variant detection and assembly of a human genome. *Nat. Biotechnol.* **37**, 1155–1162 (2019).

122. X. Vekemans, V. Castric, H. Hipperson, N. A. Müller, H. Westerdahl, Q. Cronk, Whole-genome sequencing and genome regions of special interest: Lessons from major histocompatibility complex, sex determination, and plant self-incompatibility. *Mol. Ecol.* **30**, 6072–6086 (2021).

123. M. P. Hogan, A. C. Whittington, M. B. Broe, M. J. Ward, H. L. Gibbs, D. R. Rokyta, The chemosensory repertoire of the eastern diamondback rattlesnake (*Crotalus adamanteus*) reveals complementary genetics of olfactory and vomeronasal-type receptors. *J. Mol. Evol.* **89**, 313–328 (2021).

124. T. Houwaart, S. Scholz, N. R. Pollock, W. H. Palmer, K. M. Kichula, D. Strelow, D. B. Le, D. Belick, L. Hülse, T. Lautwein, T. Wachtmeister, T. E. Wollenweber, B. Henrich, K. Köhrer, P. Parham, L. A. Guethlein, P. J. Norman, A. T. Dilthey, Complete sequences of six major histocompatibility complex haplotypes, including all the major MHC class II structures. *HLA* **102**, 28–43 (2023).

125. D. Stanojevic, D. Lin, S. Nurk, P. Florez De Sessions, M. Sikic, Telomere-to-Telomere Phased Genome Assembly Using HERRO-Corrected Simplex Nanopore Reads. bioRxiv. 594796 [Preprint] (2024).

126. W. Shen, B. Sipos, L. Zhao, SeqKit2: A Swiss army knife for sequence and alignment processing. *iMeta* **3**, e191 (2024).

127. S. S. Kamath, M. Bindra, D. Pal, C. Jain, Telomere-to-telomere assembly by preserving contained reads. *Genome Res.* **34**, 1908–1918 (2024).

128. Md. Vasimuddin, S. Misra, H. Li, S. Aluru, "Efficient architecture-aware acceleration of BWA-MEM for multicore systems" in *2019 IEEE International Parallel and Distributed Processing Symposium (IPDPS)* (IEEE, 2019), pp. 314–324; https://ieeexplore.ieee.org/document/8820962/.

129. C. Zhou, S. A. McCarthy, R. Durbin, YaHS: Yet another Hi-C scaffolding tool. *Bioinformatics* **39**, btac808 (2023).

130. O. Dudchenko, S. S. Batra, A. D. Omer, S. K. Nyquist, M. Hoeger, N. C. Durand, M. S. Shamim, I. Machol, E. S. Lander, A. P. Aiden, E. L. Aiden, De novo assembly of the *Aedes aegypti* genome using Hi-C yields chromosome-length scaffolds. *Science* **356**, 92–95 (2017).

131. M. Seppey, M. Manni, E. M. Zdobnov, "BUSCO: Assessing genome assembly and annotation completeness" in *Gene Prediction*, M. Kollmar, Ed. (Springer, 2019), vol. 1962, pp. 227–245; http://link.springer.com/10.1007/978-1-4939-9173-0_14.

132. O. Nishimura, Y. Hara, S. Kuraku, gVolante for standardizing completeness assessment of genome and transcriptome assemblies. *Bioinformatics* **33**, 3635–3637 (2017).

133. M. R. Brown, P. Manuel Gonzalez De La Rosa, M., Blaxter, tidk: A toolkit to rapidly identify telomeric repeats from genomic datasets. *Bioinformatics* **41**, btaf049 (2025).

134. J. M. Flynn, R. Hubley, C. Goubert, J. Rosen, A. G. Clark, C. Feschotte, A. F. Smit, RepeatModeler2 for automated genomic discovery of transposable element families. *Proc. Natl. Acad. Sci. U.S.A.* **117**, 9451–9457 (2020).

135. J. Storer, R. Hubley, J. Rosen, T. J. Wheeler, A. F. Smit, The Dfam community resource of transposable element families, sequence models, and genome annotations. *Mob. DNA* **12**, 2 (2021).

136. M. Tarailo-Graovac, N. Chen, Using RepeatMasker to Identify Repetitive Elements in Genomic Sequences. *Curr. Protoc. Bioinformatics* **4**, 4.10.1–4.10.14 (2009).

137. J. Keilwagen, F. Hartung, J. Grau, "GeMoMa: Homology-based gene prediction utilizing intron position conservation and RNA-seq data" in *Gene Prediction*, M. Kollmar, Ed. (Springer, 2019), vol. 1962, pp. 161–177; http://link.springer.com/10.1007/978-1-4939-9173-0_9.

138. C. Peng, D.-D. Wu, J.-L. Ren, Z.-L. Peng, Z. Ma, W. Wu, Y. Lv, Z. Wang, C. Deng, K. Jiang, C. L. Parkinson, Y. Qi, Z.-Y. Zhang, J.-T. Li, Large-scale snake genome analyses provide insights into vertebrate development. *Cell* **186**, 2959–2976.e22 (2023).

139. L. Gabriel, T. Brůna, K. J. Hoff, M. Ebel, A. Lomsadze, M. Borodovsky, M. Stanke, BRAKER3: Fully automated genome annotation using RNA-seq and protein evidence with GeneMark-ETP, AUGUSTUS, and TSEBRA. *Genome Res.* **34**, 769–777 (2024).

140. B. J. Haas, S. L. Salzberg, W. Zhu, M. Pertea, J. E. Allen, J. Orvis, O. White, C. R. Buell, J. R. Wortman, Automated eukaryotic gene structure annotation using EVidenceModeler and the Program to Assemble Spliced Alignments. *Genome Biol.* **9**, R7 (2008).

141. H. Li, Minimap2: Pairwise alignment for nucleotide sequences. *Bioinformatics* **34**, 3094–3100 (2018).

142. M. M. Malmberg, G. C. Spangenberg, H. D. Daetwyler, N. O. I. Cogan, Assessment of low-coverage nanopore long read sequencing for SNP genotyping in doubled haploid canola (Brassica napus L.). *Sci. Rep.* **9**, 8688 (2019).

143. M. M. Malmberg, D. M. Barbulescu, M. C. Drayton, M. Shinozuka, P. Thakur, Y. O. Ogaji, G. C. Spangenberg, H. D. Daetwyler, N. O. I. Cogan, Evaluation and recommendations for routine genotyping using skim whole genome re-sequencing in canola. *Front. Plant Sci.* **9**, 1809 (2018).

144. C. C. Chang, C. C. Chow, L. C. Tellier, S. Vattikuti, S. M. Purcell, J. J. Lee, Second-generation PLINK: Rising to the challenge of larger and richer datasets. *Gigascience* **4**, 7 (2015).

145. R. Meyermans, W. Gorssen, N. Buys, S. Janssens, How to study runs of homozygosity using PLINK? A guide for analyzing medium density SNP data in livestock and pet species. *BMC Genomics* **21**, 94 (2020).

146. L. Fang, J. Hu, D. Wang, K. Wang, NextSV: A meta-caller for structural variants from low-coverage long-read sequencing data. *BMC Bioinformatics* **19**, 180 (2018).

147. Y. Guo, M. Jayakodi, A. Himmelbach, E. Ben-Yosef, U. Davidovich, M. David, A. Hartmann-Shenkman, M. Kislev, T. Fahima, V. J. Schuenemann, E. Reiter, J. Krause, B. J.

Steffenson, N. Stein, E. Weiss, M. Mascher, A haplotype-based evolutionary history of barley domestication. *Nature* **647**, 680–688 (2025).

148. C. Jain, A. Rhie, N. F. Hansen, S. Koren, A. M. Phillippy, Long-read mapping to repetitive reference sequences using Winnowmap2. *Nat. Methods* **19**, 705–710 (2022).

149. M. Smolka, L. F. Paulin, C. M. Grochowski, D. W. Horner, M. Mahmoud, S. Behera, E. Kalef-Ezra, M. Gandhi, K. Hong, D. Pehlivan, S. W. Scholz, C. M. B. Carvalho, C. Proukakis, F. J. Sedlazeck, Detection of mosaic and population-level structural variants with Sniffles2. *Nat. Biotechnol.* **42**, 1571–1580 (2024).

150. T. Jiang, Y. Liu, Y. Jiang, J. Li, Y. Gao, Z. Cui, Y. Liu, B. Liu, Y. Wang, Long-read-based human genomic structural variation detection with cuteSV. *Genome Biol.* **21**, 189 (2020).

151. D. C. Jeffares, C. Jolly, M. Hoti, D. Speed, L. Shaw, C. Rallis, F. Balloux, C. Dessimoz, J. Bähler, F. J. Sedlazeck, Transient structural variations have strong effects on quantitative traits and reproductive isolation in fission yeast. *Nat. Commun.* **8**, 14061 (2017).

152. D. V. Klopfenstein, L. Zhang, B. S. Pedersen, F. Ramírez, A. Warwick Vesztrocy, A. Naldi, C. J. Mungall, J. M. Yunes, O. Botvinnik, M. Weigel, W. Dampier, C. Dessimoz, P. Flick, H. Tang, GOATOOLS: A Python library for Gene Ontology analyses. *Sci. Rep.* **8**, 10872 (2018).

153. F. Cabanettes, C. Klopp, D-GENIES: Dot plot large genomes in an interactive, efficient and simple way. *PeerJ* **6**, e4958 (2018).

154. M. Alonge, L. Lebeigle, M. Kirsche, K. Jenike, S. Ou, S. Aganezov, X. Wang, Z. B. Lippman, M. C. Schatz, S. Soyk, Automated assembly scaffolding using RagTag elevates a new tomato system for high-throughput genome editing. *Genome Biol.* **23**, 258 (2022).

155. H. Li, B. Handsaker, A. Wysoker, T. Fennell, J. Ruan, N. Homer, G. Marth, G. Abecasis, R. Durbin, 1000 Genome Project Data Processing Subgroup, *The Sequence Alignment/Map format and SAMtools*, *Bioinformatics* **25**, 2078–2079 (2009).

156. A. R. Quinlan, I. M. Hall, BEDTools: A flexible suite of utilities for comparing genomic features. *Bioinformatics* **26**, 841–842 (2010).

157. J. T. Lovell, A. Sreedasyam, M. E. Schranz, M. Wilson, J. W. Carlson, A. Harkess, D. Emms, D. M. Goodstein, J. Schmutz, GENESPACE tracks regions of interest and gene copy number variation across multiple genomes. *eLife* **11**, e78526 (2022).

158. J. Dainat, D. Hereñú, E. Davis, K. Crouch, LucileSol, Pascal-Git, Tayyrov, NBISweden/AGAT: AGAT-v0.9.1, version v0.9.1 (Zenodo, 2022); https://doi.org/10.5281/ZENODO.3552717.

159. B. W. Perry, D. C. Card, J. W. McGlothlin, G. I. M. Pasquesi, R. H. Adams, D. R. Schield, N. R. Hales, A. B. Corbin, J. P. Demuth, F. G. Hoffmann, M. W. Vandewege, R. K. Schott, N. Bhattacharyya, B. S. W. Chang, N. R. Casewell, G. Whiteley, J. Reyes-Velasco, S. P. Mackessy, T. Gamble, K. B. Storey, K. K. Biggar, C. N. Passow, C.-H. Kuo, S. E. McGaugh, A. M. Bronikowski, A. P. J. De Koning, S. V. Edwards, M. E. Pfrender, P. Minx, E. D. Brodie, E. D. Brodie, W. C. Warren, T. A. Castoe, Molecular adaptations for sensing and securing prey and insight into amniote genome diversity from the garter snake genome. *Genome Biol. Evol.* **10**, 2110–2129 (2018).

160. B. S. Pedersen, A. R. Quinlan, Mosdepth: Quick coverage calculation for genomes and exomes. *Bioinformatics* **34**, 867–868 (2018).

161. K. Suryamohan, S. P. Krishnankutty, J. Guillory, M. Jevit, M. S. Schröder, M. Wu, B. Kuriakose, O. K. Mathew, R. C. Perumal, I. Koludarov, L. D. Goldstein, K. Senger, M. D. Dixon, D. Velayutham, D. Vargas, S. Chaudhuri, M. Muraleedharan, R. Goel, Y.-J. J. Chen, A. Ratan, P. Liu, B. Faherty, G. De La Rosa, H. Shibata, M. Baca, M. Sagolla, J. Ziai, G. A. Wright, D. Vucic, S. Mohan, A. Antony, J. Stinson, D. S. Kirkpatrick, R. N. Hannoush, S. Durinck, Z. Modrusan, E. W. Stawiski, K. Wiley, T. Raudsepp, R. M. Kini, A. Zachariah, S. Seshagiri, The Indian cobra reference genome and transcriptome enables comprehensive identification of venom toxins. *Nat. Genet.* **52**, 106–117 (2020).

162. Z.-Y. Zhang, Y. Lv, W. Wu, C. Yan, C.-Y. Tang, C. Peng, J.-T. Li, The structural and functional divergence of a neglected three-finger toxin subfamily in lethal elapids. *Cell Rep.* **40**, 111079 (2022).

163. C. Yan, W. Wu, W. Dong, B. Zhu, J. Chang, Y. Lv, S. Yang, J.-T. Li, Temperature acclimation in hot-spring snakes and the convergence of cold response. *Int. J. Hydrogen Energ.* **3**, 100295 (2022).

164. C.-Y. Tang, X. Zhang, X. Xu, S. Sun, C. Peng, M.-H. Song, C. Yan, H. Sun, M. Liu, L. Xie, S.-J. Luo, J.-T. Li, Genetic mapping and molecular mechanism behind color variation in the Asian vine snake. *Genome Biol.* **24**, 46 (2023).
